# Much improved thin-film photodiodes with novel organic interlayer

**DOI:** 10.1093/nsr/nwag013

**Published:** 2026-01-09

**Authors:** Karl Leo

**Affiliations:** Dresden Integrated Center for Applied Physics and Photonics and Institute for Applied Physics, Department of Physics, Technische Universität Dresden, Germany

Optoelectronic devices realized with novel semiconductor materials such as quantum dots or organic semiconductors enable attractive new applications. One example is the success in OLED display application, reaching double-digit multibillion markets [1]. However, there are many other exciting applications of nonconventional semiconductors which still require significantly improved device performance in order to achieve application impact. Among those are thin-film photodetectors, which have many advantages like easy spectral tunability, flexibility, and low-cost production, but suffer from unwanted effects such as high dark currents which reduce detectivity [2]. This hinders their widespread use in applications such as telecommunications, night vision, environmental sensing, and biomedical imaging.

In a recent article, Cao *et al*. [3] present a new class of hybrid ionic-electronic semiconductors which significantly improve the performance of solution-processed short-wave infrared (SWIR) quantum dot photodetectors, by creating improved interfaces between the device organic layers and electrodes. The key innovation lies in engineering an ionic component within an electronic semiconductor matrix, creating a material which has a dual advantage when applied as an interlayer between electrode and device active layers: first, it modifies the energy landscape by adjusting the electrode work function (WF) to suppress reverse injection currents at the contact. Second, it suppresses defects which reduces unwanted reverse current generation at the trap states associated with these defects. Thus, this engineered interface efficiently suppresses the reverse dark current, which is usually the dominating source of noise in photodetectors. Since the detectivity is basically defined as the ratio of signal current to noise, it can easily grow by orders of magnitude when the dark currents are suppressed by the same ratio [4].

**Figure 1.**
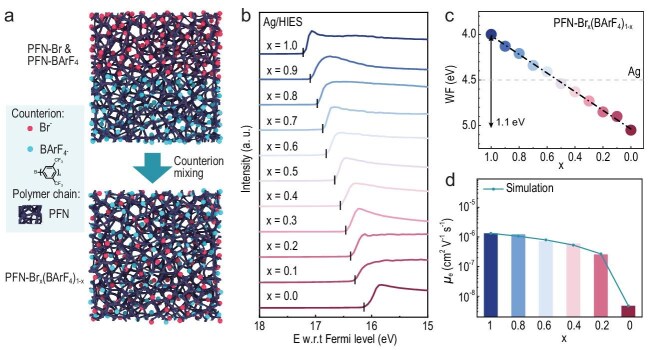
Characteristics of the new interlayer materials. (a) Schematic diagram of the formation mechanism of the materials, with PFN-Br_x_(BArF_4_)_1−x_ as an example. (b) UPS cutoff edge of Ag electrode modified with PFN-Br_x_(BArF_4_)_1−x_, illustrating shifts in the cutoff edge with varying x (Br^-^ proportion). (c) WF of Ag electrode modified with the interlayer, derived from UPS measurements. The dashed grey line represents the WF of unmodified Ag. (d) The intrinsic electron mobility of PFN-Br_x_(BArF_4_)_1−x_ films.

The work is based on conjugated polyelectrolytes (CPEs), which can modify the WF of an electrode by creating a defined electric dipole moment at the interface to the metal. By using a clever blending strategy with two different counterions with opposite dipole effect, Cao *et al*. manage to precisely control the WF modification by adjusting the counterion ratio. Through photoemission spectroscopy, they prove that the CPE layer enables precise WF modulation of an Ag electrode over the broad range of 4.0 to 5.1 eV, with a tunable resolution of 0.1 eV (Fig. 1). This property alone is extremely useful and can find application in almost all thin-film electronic devices.

The authors show that beyond WF modification, there are more subtle physics involved. First, they show that the electron transfer rate at the contact is reduced by coupling to the vibrations of the CPE layer. Second, they prove by capacitance measurements that the trap density of states at the interface is significantly reduced, thus preventing additional trap-mediated dark currents. Both effects are very useful for device application and motivate further studies to better understand the microscopic mechanisms behind them.

In summary, Cao *et al*. [3] have presented an interesting new approach to tune the WF of electrons, with additional benefits besides simple energy adjustment. The novel conjugated polyelectrolytes are solution processable and thus suitable for cost-effective, low-temperature deposition methods such as printing or coating. In Ref. [3], the technique is successfully applied to photodetectors, but it is highly likely that it will prove its usefulness in many other thin-film devices.
